# Machine-learning predictions for acute kidney injuries after coronary artery bypass grafting: a real-life muticenter retrospective cohort study

**DOI:** 10.1186/s12911-023-02376-0

**Published:** 2023-11-23

**Authors:** Tianchen Jia, Kai Xu, Yun Bai, Mengwei Lv, Lingtong Shan, Wei Li, Xiaobin Zhang, Zhi Li, Zhenhua Wang, Xin Zhao, Mingliang Li, Yangyang Zhang

**Affiliations:** 1 College of Information Science, Shanghai Ocean University, Shanghai, P.R. China; 2 Department of Cardiovascular Surgery, Qilu Hospital of Shandong University, Jinan, Shandong P.R. China; 3 Department of Thoracic Surgery, Xuzhou Cancer Hospital, Xuzhou, P.R. China; 4 Department of Thoracic Surgery, Sheyang County People’s Hospital, Yancheng, P.R. China; 5 Department of Cardiovascular Surgery, Shanghai Chest Hospital, Shanghai Jiao Tong University School of Medicine, 241 Huaihai West Road, Shanghai, 200120 China; 6 Department of Cardiovascular Surgery, Jiangsu Province Hospital, the First Affiliated Hospital of Nanjing Medical University, Nanjing, P.R. China; 7 Department of Cardiovascular Surgery, The General Hospital of Ningxia Medical University, Yinchuan, Ningxia P.R. China

**Keywords:** Acute kidney injuries, Prediction model, Machine learning, Coronary artery bypass grafting

## Abstract

**Background:**

Acute kidney injury (AKI) after coronary artery bypass grafting (CABG) surgery is associated with poor outcomes. The objective of this study was to apply a new machine learning (ML) method to establish prediction models of AKI after CABG.

**Methods:**

**A total of** 2,780 patients from two medical centers in East China who underwent primary isolated CABG were enrolled. The dataset was randomly divided for model training (80%) and model testing (20%). Four ML models based on LightGBM, Support vector machine (SVM), Softmax and random forest (RF) algorithms respectively were established in Python. A total of 2,051 patients from two other medical centers were assigned to an external validation group to verify the performances of the ML prediction models. The models were evaluated using the area under the receiver operating characteristics curve (AUC), Hosmer-Lemeshow goodness-of-fit statistic, Bland-Altman plots, and decision curve analysis. The outcome of the LightGBM model was interpreted using SHapley Additive exPlanations (SHAP).

**Results:**

The incidence of postoperative AKI in the modeling group was 13.4%. Similarly, the incidence of postoperative AKI of the two medical centers in the external validation group was 8.2% and 13.6% respectively. LightGBM performed the best in predicting, with an AUC of 0.8027 in internal validation group and 0.8798 and 0.7801 in the external validation group. The SHAP revealed the top 20 predictors of postoperative AKI ranked according to the importance, and the top three features on prediction were the serum creatinine in the first 24 h after operation, the last preoperative Scr level, and body surface area.

**Conclusion:**

This study provides a LightGBM predictive model that can make accurate predictions for AKI after CABG surgery. The LightGBM model shows good predictive ability in both internal and external validation. It can help cardiac surgeons identify high-risk patients who may experience AKI after CABG surgery.

## Introduction

Coronary artery bypass grafting (CABG) surgery is currently the main clinical treatment for serious coronary heart disease (CHD) and is increasingly applied to patients worldwide. Postoperative complications of CABG include perioperative myocardial ischemia, arrhythmias, acute kidney injury (AKI), neurological complications, and bleeding. AKI is a common complication after cardiac surgery with a typical incidence of 15-30% [1–3]. In practice, failure to identify patients at high risk of AKI in the early stages following CABG and to pre-empt treatment may cause AKI to develop to chronic renal failure or even end-stage renal disease, ultimately increasing the risk of death. The high postoperative incidence and associated high mortality make AKI a great concern of cardiac surgery. However, the pathogenesis of AKI after CABG is very complex and not completely understood [4]. As such, it is urgent to identify the risk factors of postoperative AKI and to explore prediction models of AKI after CABG.

Machine learning (ML) is a branch of artificial intelligence that has been increasingly used in various fields to analyze massive data. With the development of information technology recently, hospital electronic medical record systems generate huge amounts of data yearly, which have led many health and biomedical researchers to apply ML, especially prediction models, to extract valuable insights from the growing biomedical database. Unlike traditional statistical methods that use selected variables for further calculations, ML can easily combine a large number of variables using a computer algorithm, which improves the forecasting accuracy. ML-based models outperform traditional statistical models based on the logistic regression algorithm [5, 6]. However, predicting AKI in CABG patients with ML methods has not attracted much attention from researchers.

This study aims to use ML based on Light gradient boosting machine (Light GBM), Support vector machines (SVM), Softmax and random forest (RF) to establish models enabling early and effective prediction of AKI after CABG, which are needed to identify high-risk patients and have practical guiding significance to clinical decision-making.

## Methods

### Study population

This project is a multi-center retrospective study. A total of 4,170 patients undergoing CABG from two medical centers of East China (the Jiangsu Province Hospital Affiliated to Nanjing Medical University, JSPH, and Shanghai Chest Hospital Affiliated to Shanghai Jiaotong University, SHCH) were enrolled. The inclusion criterion was: CABG surgery for severe CHD. The exclusion criteria were: (1) age less than 18 years; (2) redo CABG surgery; (3) combined with other cardiac procedures (e.g., valve, ventricular aneurysm, ventricular septum); (4) absence of perioperative medical records; (5) preoperative chronic renal failure; (6) preoperative hemodialysis treatment. Ultimately, 2,780 patients were enrolled and randomly assigned to a model training group and an internal validation group at a ratio of 8:2.

**Fig. 1:**
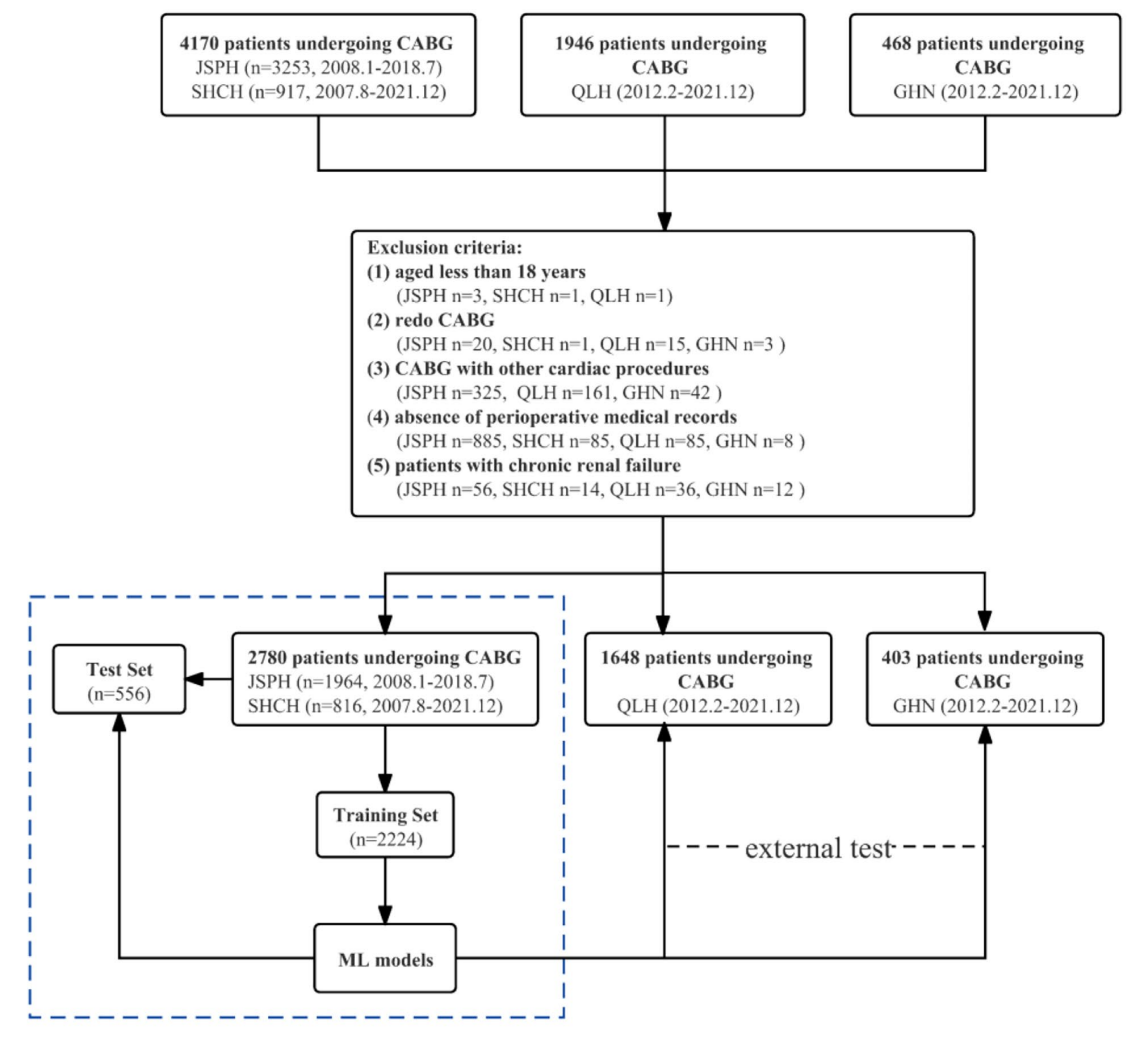
Flowchart of patient selection. (JSPH: Jiangsu Province Hospital, the First Affiliated Hospital of Nanjing Medical University; SHCH: Shanghai Chest Hospital of Shanghai Jiao Tong University; QLH: Qilu Hospital of Shandong University; GHN: General Hospital of Ningxia Medical University; CABG: Coronary artery bypass grafting)

Another 2,414 patients from two medical centers (Qilu Hospital of Shandong University, QLH, General Hospital of Ningxia Medical University, GHN) were also enrolled, which were more than 500 km apart from the previous medical centers, located in North China and North West China respectively. According to the same inclusion and exclusion criteria, 2,051 patients were assigned to an external validation group to verify the performances of the ML models. The patients screening process was shown in Fig. 1.

### Informed consent

This study was conducted in accordance with the Declaration of Helsinki (revised 2013). Approval was obtained from the hospital ethics committees (No. 2023-SR-229 from JSPH; No. IS23002 from SHCH; No. KYLL-202204-016 from QLH; No. KYLL20230330 from GHN). Written informed consent was waived for this retrospective analysis because all the protected health information was anonymized.

### Definition and endpoints

According to the Kidney Disease Improving Global Outcome (KDIGO) clinical practice guidelines [7], AKI is defined when any of the following three criteria is met: increase in serum creatinine (Scr) by ≥ 0.3 mg/dl (≥ 26.5 umol/l) within 48 h or an increase in Scr by ≥ 1.5 times from the baseline, which is known or presumed to have occurred within the preceding 7 days; or a urine volume < 0.5 ml/kg/h for 6 h. Baseline creatinine level was defined as the preoperative value obtained closest to the date of the operation (within 48 h before the operation). Because of the application of diuretics and the difficulty in collecting clinical records, urine volume was not used to diagnose preoperative AKI. The preoperative estimated glomerular filtration rate (eGFR) was calculated with the CKD-EPIscr Eq. [8]. Proposed KDIGO staging of AKI was as follows: stage 1 was defined as increase in Scr by 1.5–1.9 times from the baseline or ≥ 0.3 mg/dl (26.5 µmol/l); Stage 2 was defined as increase in Scr by 2.0-2.9 times from the baseline; Stage 3 was defined as increase in Scr by 3 times from the baseline or ≥ 4.0 mg/dl (353.6 µmol/l) or initiation of renal replacement therapy [9].

The study outcome was the occurrence of postoperative AKI.

### Feature selection

Features were selected by referring to the European System for Cardiac Operative Risk Evaluation II (EuroSCORE II), which was improved on the basis of EuroSCORE. It not only better predicts the mortality risk of CABG, but also applies to Chinese patients [10–14]. Therefore, to comprehensively reflect the specific circumstances of patients, the risk factors selected here were based on relevant EuroSCORE II items. A total of 26 variables were collected according to relevant studies and clinical availability (Table 1). All data for the included variables were extracted from inpatient electronic medical records.

**Table 1:** Baseline clinical characteristics of modeling group and validation groups

	Modeling Group (n = 2224)	Internal Validation Group (n = 556)	*P* value	External Validation Group			
				QLH(n = 1648)	*P* value	GHN(n = 403)	*P* value
Age(y)	66(12)	66(11)	0.053	66(12)	<0.001	63(10)	<0.001
Gender(male) (n, %)	1694(76.17)	426(76.61)	0.824	1181(71.66)	0.002	275(68.23)	0.001
Weight (kg)	68.00(14.00)	69.00(14.00)	0.061	70.00(15.00)	<0.001	69.01(14.00)	<0.001
Height (cm)	168.00(12.00)	168.00(12.00)	0.163	170.00(12.00)	<0.001	166.26(12.00)	<0.001
BMI (kg/m^2^)	24.65(3.85)	24.91(3.44)	0.611	24.65(4.44)	0.091	24.95(4.41)	0.050
BSA (m^2^)	1.86(0.22)	1.86(0.21)	0.886	1.90(0.22)	<0.001	1.86(0.22)	0.063
Morbid obesity (n, %)	113(5.10)	20(3.60)	0.143	101(6.12)	0.158	18(4.47)	0.602
NYHA IV (n, %)	52(2.33)	6(1.08)	0.063	83(5.04)	<0.001	16(3.17)	0.058
CAD classification			0.001		<0.001		<0.001
Stable angina (n, %)	559(25.13)	167(30.03)		111(6.74)		36(8.68)	
Unstable angina (n, %)	1349(60.66)	296(53.23)		1483(89.99)		225(55.83)	
AMI (n, %)	274(12.32)	94(16.91)		52(3.16)		66(16.38)	
Hypertension (n, %)	1532(68.89)	411(73.92)	0.021	1023(62.08)	<0.001	253(62.78)	0.016
Diabetes (n, %)	747(29.51)	211(37.95)	0.053	562(34.10)	0.738	152(37.72)	0.108
Cerebrovascular disease (n, %)	340(15.29)	60(10.79)	0.007	175(10.62)	<0.001	36(8.93)	0.001
Preoperative Scr (µmol/l)	76.70(26.00)	80.00(26.00)	0.514	72.00(20.00)	<0.001	74.63(24.30)	<0.001
Preoperative eGFR (mL/min/1.73 m^2^)	77.32(34.11)	75.26(33.16)	0.047	84.63(30.31)	<0.001	90.11(34.69)	<0.001
Preoperative LVEF (%)	62.00(8.88)	62.00(8.88)	0.071	60.00(13.00)	<0.001	59.13(16.99)	<0.001
Number of diseased vessels (n, %)			<0.001		<0.001		0.022
1	106(4.77)	40(7.19)		15(0.91)		9(2.23)	
2	244(10.97)	87(15.64)		176(10.68)		35(8.68)	
3	1874(84.26)	429(77.16)		1457(88.41)		359(89.08)	
Peripheral vascular disease (n, %)	206(9.26)	143(25.72)	<0.001	40(2.42)	<0.001	141(34.99)	<0.001
Surgical status (n, %)			0.217		<0.001		<0.001
Emergency	64(2.89)	10(1.80)		1(0.06)		1(0.25)	
Rescue	22(0.98)	3(0.53)		2(0.12)		0(0.00)	
Valvular disease (n, %)	271(12.18)	66(11.87)	0.839	50(3.03)	<0.005	27(6.70)	0.001
IABP implantation (n, %)	73(3.28)	26(4.68)	0.113	55(3.34)	0.925	17(4.22)	0.342
COPD (n, %)	111(4.99)	38(6.83)	0.084	9(0.55)	<0.001	28(6.95)	0.106
Atrial fibrillation (n, %)	71(3.19)	21(3.78)	0.491	30(1.82)	0.006	13(3.23)	0.972
Pulmonary hypertension (n, %)	95(4.27)	43(7.73)	0.001	95(5.76)	0.084	3(0.74)	0.001
Previous PCI (n, %)	209(9.40)	36(6.47)	0.030	85(5.16)	<0.001	40(9.93)	0.739
Bypass graft number(n)	3.00(1.00)	3.00(1.00)		3.00(1.00)		3.00(1.00)	
Cardiopulmonary bypass (n, %)	129(5.80)	61(10.97)	<0.001	121(7.34)	0.054	4(0.99)	<0.001
AKI (n, %)	352(15.82)	22(3.96)	<0.001	135(8.19)	<0.001	55(13.64)	0.266
Stage 1	233(10.47)	18(3.23)		97(5.89)		37(9.18)	
Stage 2	73(3.28)	2(0.36)		29(1.76)		12(2.98)	
Stage 3	46(2.07)	2(0.36)		9(0.55)		6(1.49)	

Abbreviation: QLH, Qilu Hospital of Shandong University; GHN, General Hospital of Ningxia Medical University; BMI, body mass index; BSA, body surface area; NYHA, New York heart association; CAD, coronary artery disease; AMI, acute myocardial infarction; Scr, Serum creatinine; eGFR, estimated glomerular filtration rate; LVEF, left ventricular ejection fraction; IABP, intra-aortic balloon pump; COPD, chronic obstructive pulmonary disease; PCI, percutaneous coronary intervention; AKI, acute kidney injuries

### Machine learning modeling

#### Light gradient boosting machine (LightGBM)

In 2017, a team at Microsoft introduced a new efficient gradient boosting algorithm based on decision trees, named LightGBM. Building upon the foundation of GBDT, LightGBM incorporates the Histogram algorithm and a leaf-wise growth strategy [15].

**Fig. 2:**
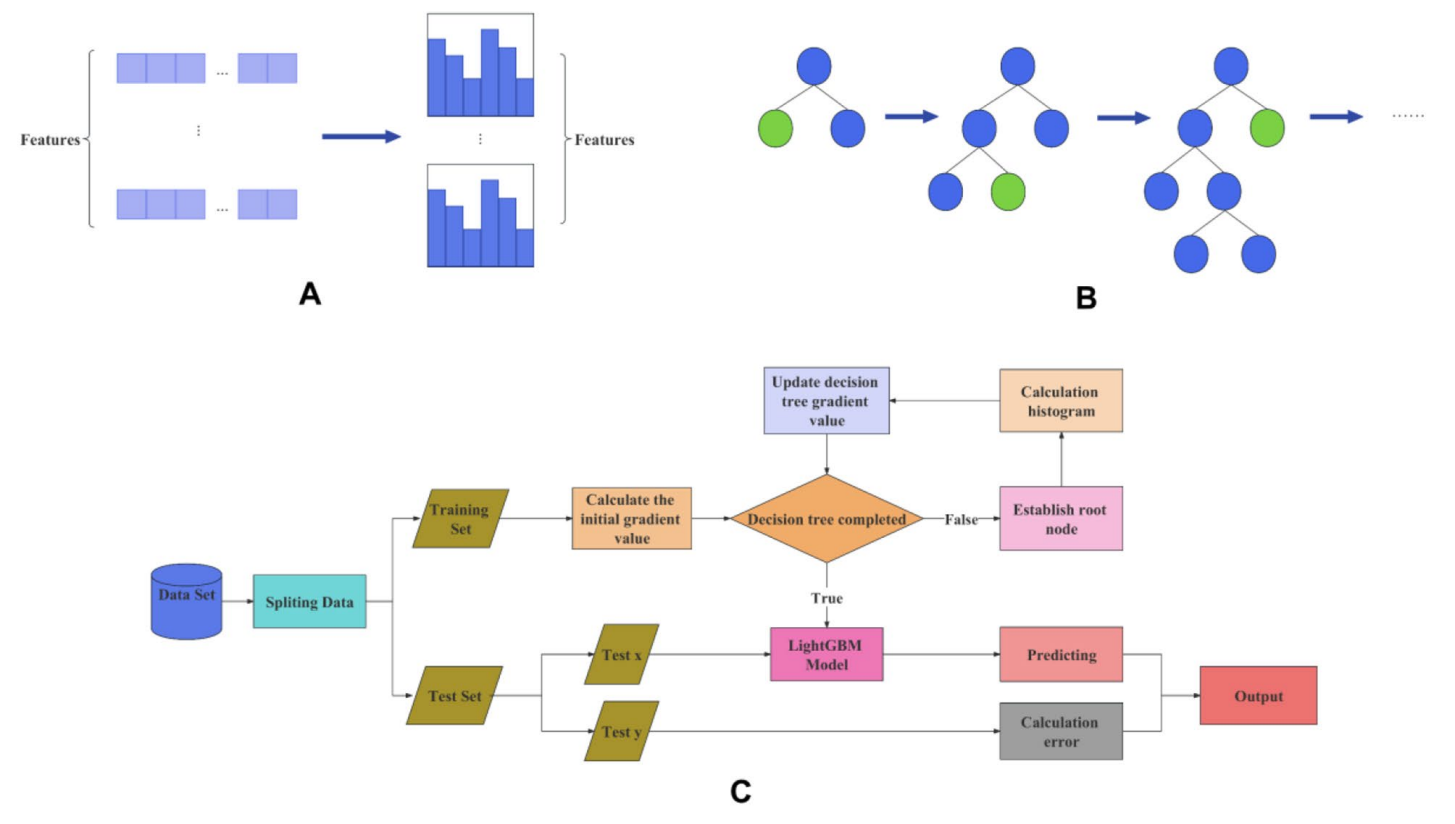
The introduction of (**A**) histogram algorithm, (**B**) leaf-wise strategy and (**C**) the specific process of LightGBM

The basic idea of Histogram algorithm is illustrated in Fig. 2 (A) and the process is as follows. For continuous features, convert them into N distinct values, and then build a histogram that spans these N values. In the case of discrete features, place each unique value into a specific bin. If the number of unique values exceeds the available bins, less frequent values are ignored. When traversing the data, the statistical information is accumulated in the histogram using the discretized values as indices. This accumulation ensures that after one traversal, the histogram contains the necessary statistical data, which is subsequently used to determine the best split point by traversing the discretized values of the histogram.

The basic idea of the leaf-wise growth strategy is illustrated in Fig. 2 (B), the process is as follows. For each splitting process of a leaf node, identify the leaf node with the highest splitting gain in the current layer (the green node), then split it, and so on.

In the context of the relationship between the Histogram algorithm and the leaf-wise growth strategy, they work in tandem. The histogram algorithm prioritizes speed and memory efficiency, while the Leaf-wise growth strategy focuses on accuracy optimization. Together, these strategies form a harmonious partnership. And the specific process of the model was shown in Fig. 2 (C).

In addition, in this study, the SHapley Additive exPlanation (SHAP) method was used to explain the LightGBM model. SHAP, a unified approach for explaining the outcome of any machine learning model, was used to provide consistent and locally accurate attribution values for each feature within the ML model. In SHAP, all features are considered as contributors, and the model generates a prediction value (SHAP value) indicating the contribution of the feature for each prediction sample. SHAP values are very different from traditional variable screening methods (e.g., subset selection methods, coefficient compression methods, dimensionality reduction methods), which rely on model judging metrics. The SHAP value is a game-theoretic approach to interpret the output of any ML model, and interprets the model-predicted value as the sum of the inputted values of each feature, where the imputed values are the SHAP values. It has several advantages: (1) SHAP can explain the output of a single sample, not just the global importance; (2) SHAP can explain the effects of each feature on the model output, including positive and negative effects; (3) SHAP values can be used to visually interpret the output of an ML model [16].

### Support vector machines (SVMs)

Support Vector Machines (SVMs) began as binary classifiers but have since expanded to multi-class classification using strategies like “one-vs-one” (OvO) and “one-vs-rest” (OvR). In essence, SVMs find a hyperplane in the feature space that best divides data into classes. For non-linear data, the “kernel trick” allows SVMs to map data into a higher dimension, making it linearly separable.

In our work, we used hinge loss to maximize the margin between data points and the hyperplane. The kernel function, a pivotal component of SVMs, must meet the Mercer condition to ensure the kernel matrix’s positive semi-definiteness, crucial for SVM’s numerical stability. Our chosen kernel function is the standard dot product, emphasizing our model’s interpretability in multi-class scenarios [17].

### Softmax regression

Softmax regression, also known as multinomial logistic regression, distinguishes itself by employing a discriminative vector instead of the Sigmoid function found in standard logistic regression. Specifically designed for multi-class classification, this model predicts a probability for each category through a linear function. The Softmax function then steps in to convert these scores into normalized probabilities, ensuring the sum of probabilities across all classes is unity.

The primary objective of this model is to minimize the cross-entropy loss, serving as a measure of disparity between the predicted and actual probability distributions. Owing to its straightforward structure, rapid classification speed, and minimal space requirements, Softmax regression frequently serves as a benchmark model for multi-class classification problems [18].

### Random forest (RF)

Random Forest is a supervised machine learning algorithm rooted in decision tree methodologies. Unlike single decision trees, RF constructs an ensemble of trees, each representing a distinct instance of either classification or regression based on input data. For classification tasks, the RF output is the class chosen by the majority of the trees. For regression tasks, it offers the mean or average prediction of the individual trees. A unique characteristic of RF is its use of both bagging and feature randomness, ensuring an uncorrelated forest of decision trees, setting it apart from other tree-based models. This approach affords RF a flexibility and adaptability to handle nonlinear data with remarkable accuracy. While both RF and LightGBM employ decision trees as base learners, their construction, optimization, and overall methodologies differ significantly. We have chosen to evaluate both to provide a comprehensive comparison and understanding of their performances in our specific application and also make RF as a benchmark model [19].

### Statistical and technical specifications

Categorical variables were expressed as total numbers and percentages, and differences between groups were compared using χ^2^ test or Fisher’s exact test. Continuous variables were shown as mean ± standard deviation (SD) and median with 95% confidence interval (CI). Continuous variables in normal distribution and skewed distribution were analyzed with Student’s t-test and Mann–Whitney U-test respectively (*P* < 0.05 was considered significant).

Receiver’s operating characteristics (ROC) curve and the area under the ROC curve (AUC) were used to measure the discrimination ability of ML models. Sensitivity analysis was performed to examine the predictive power of ML models. The analyses included sensitivity, specificity, positive and negative predictive values.

The net reclassification index (NRI) and integrated discrimination improvement (IDI) were used to further assess the predictive power of the two models concerning postoperative AKI [20, 21]. If the values of NRI and IDI were positive, then the first model showed a positive improvement over the other model. Conversely, it implies a negative improvement.

Calibration (statistical precision) of models was analyzed by Hosmer–Lemeshow (H-L) goodness-of-fit statistic. When *P* is larger than 0.05, the predicted postoperative AKI rate and the actual postoperative AKI rate were in good agreement.

Bland-Altman plots were used to estimate the agreement of models in pairs [22]. If the difference between the two models lies within the consistency bounds, it suggests good agreement. A higher agreement between the two models means the solid line representing the mean of the differences is closer to the dashed line with a zero value. About 95% of the difference between the values of the two models falls within the range of values described by the consistency limits, indicating that the two models are in good agreement.

In addition, decision curve analysis (DCA) was performed to assess the utility of the model in decision-making by quantifying the net utility at different threshold probabilities. Clinical net benefit was defined as the minimum probability of the disease, when further intervention was warranted [23].

The current study was designed following the transparent report of a ML architecture and the Strengthening the Reporting of Observational Studies in Epidemiology (STROBE) reporting guideline [24, 25]. All statistical analyses, including descriptive statistics, inferential tests, and specific data preprocessing steps, were performed using SPSS (R23.0.0.0) and GraphPad Prism (version 8.2.1). Python (open-source Scipy python package) was utilized for developing machine learning models based on the LightGBM, SVM, Softmax, and RF algorithms.

## Results

### Baseline characteristics

The model training group included 2,224 patients total, with a mean age of 66.00 ± 12.00 years, including 1,694 men (76.17%) and 530 women (23.83%). The preoperative renal functions of all patients were generally normal, with all eGFR ≥ 30 ml/min/1.73 m^2^, mean eGFR of 77.32 ± 33.16 ml/min/1.73 m^2^. There were 556 patients in the internal validation group. Due to the random assignment, the differences in the main clinical characteristics between the two groups were not significant (Table 1).

The external validation group was formed by 1,648 patients from QLH and 403 patients from GHN. The baseline characteristics of each group were also listed in Table 1.

### Explanation of LightGBM model with the SHAP method

**Fig. 3:**
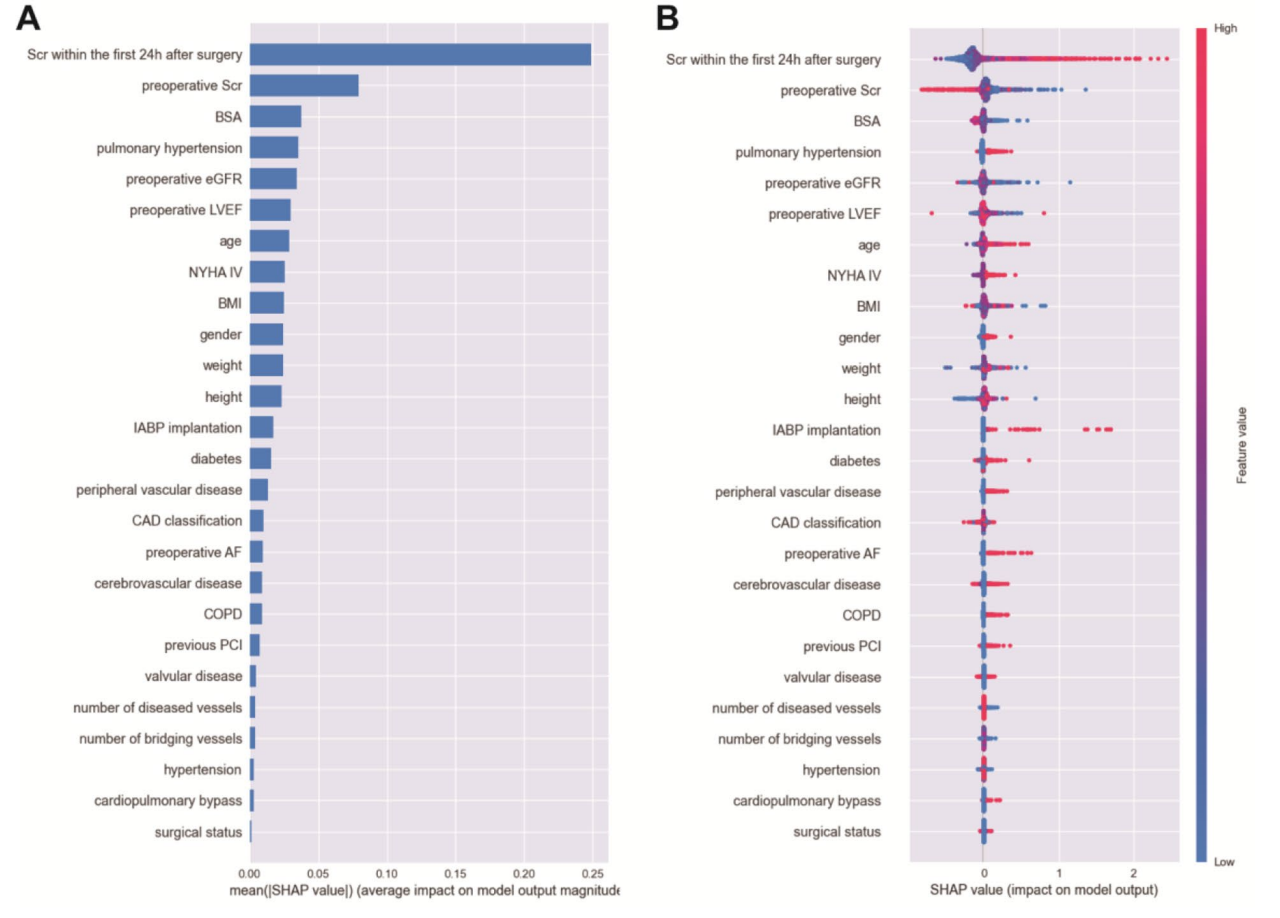
(**A**) The weights of variable importance and (**B**) the SHapley Additive exPlanation (SHAP) values of variables (Scr: serum creatinine; BSA: body surface area; eGFR: estimated glomerular filtration rate; LVEF: left ventricular ejection fraction; NYHA: New York Heart Association Class; BMI: body mass index; IABP: intra-aortic balloon pump; CAD: coronary heart disease; COPD: chronic obstructive pulmonary disease; PCI: percutaneous coronary intervention)

**Fig. 4:**
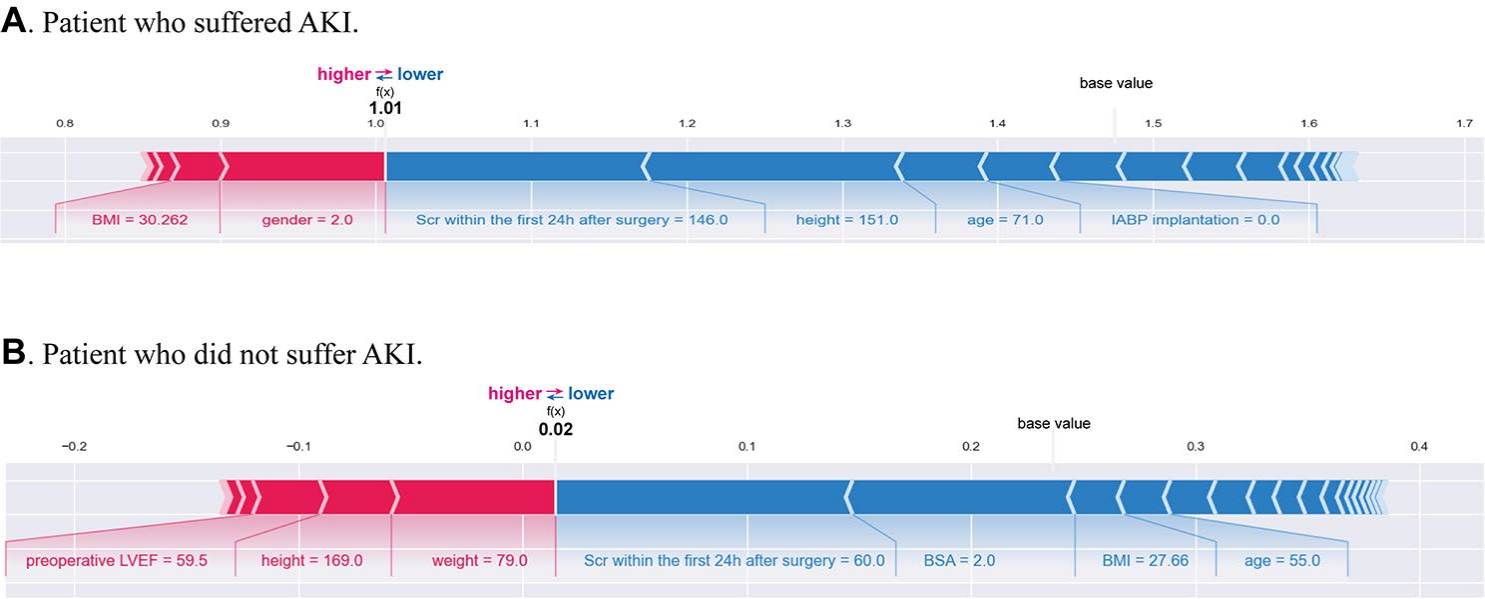
The individual SHAP force plots for patients who (**B**) did not suffer postoperative AKI and (**A**) suffered postoperative AKI

The SHAP algorithm was used to obtain the importance of each predictor variable to the outcome predicted by the LightGBM model. The top 26 importance of variables for predicting postoperative AKI were shown in Fig. 3. Scr in the first 24 h after surgery had the strongest predictive value for all prediction horizons, followed quite closely by the last preoperative Scr level, the body surface area (BSA), pulmonary hypertension, and preoperative eGFR. Furthermore, to detect the positive and negative relationships of the predictors with the target result, SHAP values were applied to uncover the postoperative AKI risk factors. As shown in plot B in Fig. 3, the horizontal location showed whether the effect of a value was associated with a higher or lower prediction, and the color indicated whether a variable was high (in red) or low (in blue) for that observation. For example, Scr in the first 24 h after surgery had a positive impact and pushed the prediction toward postoperative AKI. Figure 4 shows the individual plots for patients who did not suffer postoperative AKI and suffered postoperative AKI. The SHAP values indicated risk factors and their contribution to the prediction of postoperative AKI. Where f(x) was the predicted value of postoperative AKI, red indicated risk factors that increased postoperative AKI, and blue indicated risk factors that decreased postoperative AKI, where longer arrows indicated a greater degree of impact on postoperative AKI.

### Model evaluation

**Fig. 5:**
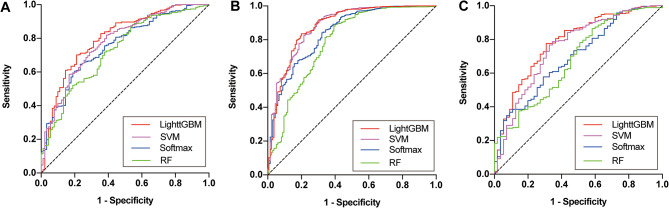
Receiver’s operating characteristic (ROC) curves of the risk evaluation models in (**A**) the internal test group and in external test groups of (**B**) QLH and (**C**) GHN (SVM: support vector machine; RF: random forest)

Within the model training group, the LightGBM, SVM, Softmax and RF models were established, and the AUCs with the internal validation group were 0.8027 (95% CI: 0.7511–0.8542), 0.7805 (0.7277–0.8333), 0.7568 (0.7042–0.8094) and 0.7292 (0.6725–0.7858), respectively (Table 2; Fig. 5). The LightGBM model showed the largest AUC with a sensitivity of 70.11% and specificity of 78.89%, while the RF model had the smallest AUC with a sensitivity of 71.89% and specificity of 62.22%. Similar results were also present in the two external validation groups. In the QLH external validation group, the LightGBM exhibited robust discriminatory power with an AUC of 0.8798 (95% CI: 0.8446–0.9150), showcasing a sensitivity of 83.54% and a specificity of 80.00%. On the other hand, the SVM model demonstrated superior performance, achieving the highest AUC of 0.8819 (0.8483–0.9156), along with a sensitivity of 91.13% and a specificity of 70.37%. In the GHN external validation group, the LightGBM model outperformed the other three models with an AUC of 0.7801 (0.7128–0.8475), demonstrating a sensitivity of 77.59% and a specificity of 69.09%. (Table 2).

**Table 2:** Performance of each model for prediction

		Cutoff	AUC	Sensitivity	Specificity	Positive predictive value	Negative predictive value
Internal validation group							
	LightGBM	0.0715	0.8027	0.7011	0.7889	0.3739	0.9790
	SVM	0.2000	0.7805	0.8197	0.6111	0.3957	0.9161
	Softmax	0.1192	0.7568	0.6459	0.7667	0.2918	0.9319
	RF	0.1754	0.7292	0.7189	0.6222	0.2995	0.9079
External validation group of QLH							
	LightGBM	0.0916	0.8798	0.8354	0.8000	0.3025	0.9791
	SVM	0.1409	0.8819	0.9113	0.7037	0.4113	0.9718
	Softmax	0.0954	0.8411	0.8414	0.6791	0.3588	0.9704
	RF	0.1592	0.7861	0.8916	0.6000	0.4239	0.9611
External validation group of GHN							
	LightGBM	0.0795	0.7801	0.7759	0.6909	0.2138	0.9070
	SVM	0.1331	0.7504	0.7874	0.6545	0.3302	0.9327
	Softmax	0.0600	0.6941	0.5776	0.7091	0.2806	0.9394
	RF	0.1932	0.6777	0.8649	0.4182	0.4600	0.9039

Abbreviation: AUC, area under the curve; SVM, support vector machine; RF, random forest

The H-L goodness-of-fit statistic was used to verify the calibration of two ML models. Only the SVM model was poorly calibrated in the internal validation group with a *P* equal to 0.046. The calibration of the remaining three models was good. In the external validation group of QLH, SVM and Softmax models were poorly calibrated with *P* less than 0.05. In comparison, LightGBM and RF models performed better with *P* above 0.05. In the external validation group of GHN, all four models showed *P* larger than 0.05.

NRI and IDI are two new evaluation metrics to assess the degree of improvement in one model over another one. Compared with SVM, RF and Softmax models, the NRIs of the LightGBM model were 0.5852 (95%CI: 0.3651–0.8053), 0.8988 (0.6860–1.1116) and 0.6847 (0.4665–0.9028) in the internal validation group, respectively; the corresponding IDIs were 0.0144 (-0.0022-0.0311), 0.1632 (0.1168–0.2097) and 0.1082 (0.0708–0.1455) respectively. The LightGBM model also showed positive gains in all two external validation groups compared to the other three ML models (Table 3).

**Table 3:** Comparison of NRI and IDI between LightGBM model and the other three models (SVM, RF, Softmax)

		NRI			IDI		
		Value	95%CI	*P*	Value	95%CI	*P*
Internal validation group							
	LightGBM-SVM	0.5852	0.3651 to 0.8053	0	0.0144	-0.0022 to 0.0311	0.0895
	LightGBM-RF	0.8988	0.686 to 1.1116	0	0.1632	0.1168 to 0.2097	0
	LightGBM-Softmax	0.6847	0.4665 to 0.9028	0	0.1082	0.0708 to 0.1455	0
External validation group of QLH							
	LightGBM-SVM	0.4398	0.2651 to 0.6146	0	0.0101	-0.0008 to 0.021	0.0696
	LightGBM-RF	1.0959	0.9317 to 1.2601	0	0.2017	0.1673 to 0.02361	0
	LightGBM-Softmax	0.3704	0.1966 to 0.5441	0	0.019	-0.0133 to 0.0513	0.2489
External validation group of GHN							
	LightGBM-SVM	0.2237	-0.004 to 0.4514	0.0541	-0.027	-0.0558 to 0.0019	0.067
	LightGBM-RF	0.6735	0.4017 to 0.9452	0	0.1012	0.0494 to 0.153	0.0001
	LightGBM-Softmax	0.4722	0.1919 to 0.7525	0.0009	0.0248	-0.0153 to 0.0649	0.2259

Abbreviation: NRI, net reclassification improvement; IDI, integrated discrimination improvement;

**Fig. 6:**
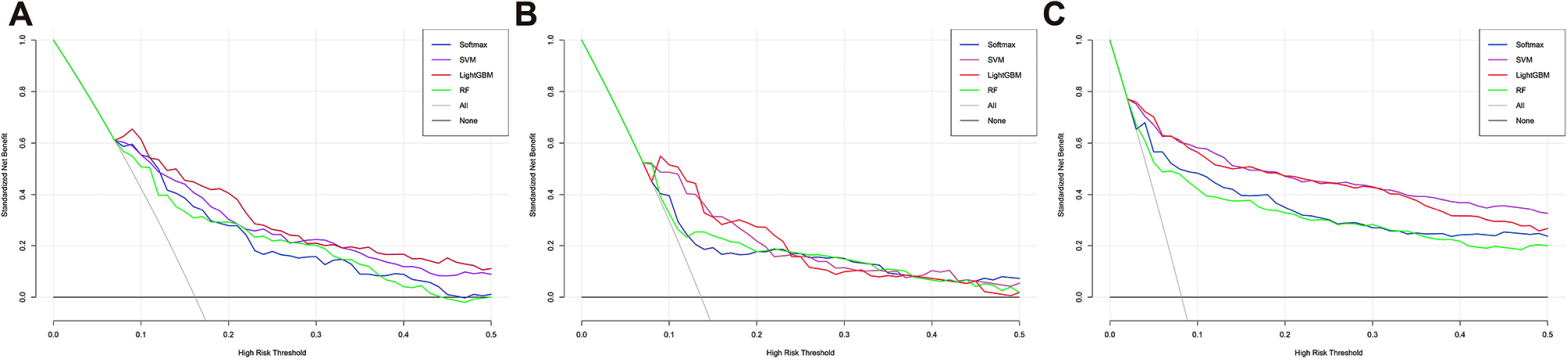
Decision curve analysis (DCA) of the four prediction models plotting the net benefit at different threshold probabilities. (**A**) DCA of the four models in the in the internal test group; (**B**) DCA of the four models in the QLH external test group; (**C**) DCA of the four models in the GHN external test group

DCA was performed for four ML models to compare the net benefit of the best model and alternative approaches for clinical decision-making. The DCA plot can visually display the clinical net benefits of the models under certain threshold probability. Because the research population varied in characteristics, treatment methods guided by any of the four ML models outperformed the default strategy of treating all or no patients. The net benefit of the LightGBM model surpassed those of the other ML models at 0–50% threshold probability in the internal validation group. In the QLH external validation group, LightGBM and SVM outperformed RF and Softmax at 9 − 21% threshold probability. Similarly, in the GHN external validation group, LightGBM and SVM have similar net benefits at 2–34% threshold probability and both outperformed the other two ML models (Fig. 6).

**Fig. 7:**
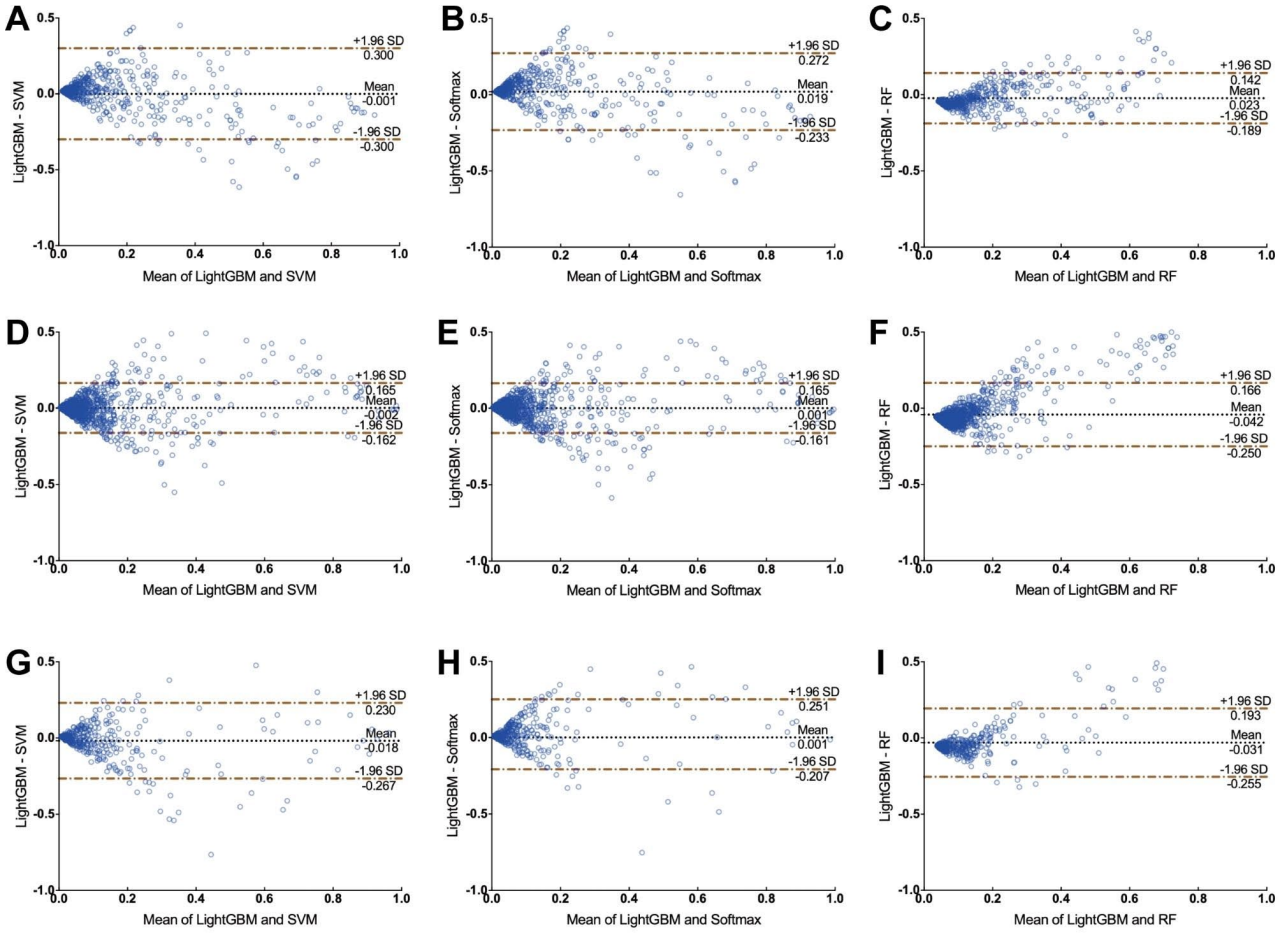
The Bland-Altman plots for postoperative AKI prediction. Consistency tests between LightGBM and (**A**) SVM, (**B**) Softmax, or (**C**) RF in the internal validation dataset; Consistency tests between LightGBM and (**D**) SVM, (**E**) Softmax, or (**F**) RF in the QLH external test group; Consistency tests between LightGBM and (**G**) SVM, (**H**) Softmax, or (**I**) RF in the GHN external test group

The Bland-Altman analysis can assess the degree of agreement between two ML models. As shown in the Bland-Altman plots, in the internal validation group, the mean of the differences between the probabilities predicted by LightGBM model and by SVM, Softmax and RF were − 0.001 ± 0.153, 0.019 ± 0.129 and 0.023 ± 0.061 respectively. Only 4%, 6% and 4% points fell outside the 95% limits of agreement (95% LoA), suggesting the agreement between the LightGBM and the other three ML models was good (Fig. 7). Similarly, the consistency between LightGBM and the other three ML models also performed relatively well in the two external validation groups (Fig. 7).

## Discussion

In this multicenter retrospective cohort study, four ML models were developed and validated using 26 features to predict AKI after CABG surgery. The LightGBM model performed the best in prediction both in the internal and external validation group, whereas the SVM model exhibited the largest AUC in the QLH external validation group. In multiaspect comprehensive evaluations, ML models especially the LightGBM model are feasible and practical in prediction of AKI after CABG surgery.

AKI is a syndrome of sudden loss of renal excretory function, and is usually accompanied by oliguria, which happens over the course of a few hours to a few days [7]. The pathogenesis of postoperative AKI is multifactorial, including ischemia–reperfusion injury, operative trauma inflammation and oxidation [26, 27]. AKI is a relatively common complication after CABG surgery. A recent retrospective study involving 32,013 patients reported a 14.3% incidence of AKI after CAGB surgery [28], which is similar to the present study. CHD is one manifestation of systemic atherosclerosis in coronary artery. Many CHD patients also suffer renal vascular diseases. Moreover, the haemodynamic instability and hypoperfusion syndrome reduced renal perfusion and raised the risk of pre-renal AKI, which, if left untreated, may lead to nephrogenic AKI [29]. Despite technological advances in renal replacement therapy, AKI is still associated with a poor outcome [30] and dramatically impacts operative mortality, intensive care unit resources, and hospital length of stay. Currently, there is no a widely-recognized model in China that can predict AKI after cardiac surgery.

In clinical practice, some medical centers had tried to establish some risk prediction models for AKI after cardiac surgery, such as the AKI following cardiac surgery score, Cleveland Clinic score, Mehta score, and simplified renal index score [31]. Nevertheless, discrimination and calibration of those models are barely satisfactory and not convincible or applicable. Therefore, AKI prediction models that are suitable for clinical practice and have predictive efficiency are urgently needed.

With advances in medical informatics, ML as a branch of artificial intelligence has become a promising tool for clinical predictive models [32, 33]. Although predictive models based on traditional statistics have been reported, ML models specifically those for AKI after CABG surgery have not been established. The fundamental distinction from ML is that the first step in traditional statistics is to build an important relationship between variables and specific outcomes. Then an equation or function that links them together is generated. This makes the predictive models based on traditional statistics understandable and more interpretable. In contrast, ML methods presuppose a meaningful relationship between a set of independent variables and the dependent variable, and then directly find the path that most strongly connects the two variables. Due to the inherent power of capturing the nonlinear relationships with ML algorithms, some cardiac surgeons advocate new ML-based models to predict cardiac surgery-associated AKI rather than traditional clinical scoring tools [34]. However, ML methods generate algorithms that are more often ‘black boxes’ of opacity to varying degrees. The nature of black-box is difficult to explain, which partially hampers their use in clinical practice.

Considering the imperative facets of model interpretability alongside its efficacy in the realm of classification, this research exercise made a deliberate selection to employ the lightGBM, SVM, Softmax, and RF algorithms for the purpose of constructing predictive models. These models are well-known for their strong predictive performance and have been widely used in various prediction tasks, including those of medical relevance [34–38].

In recent years, more and more researchers have tried to explain ML models by using the feature attribution framework of SHAP. With SHAP to explain the LightGBM model, several variables associated with AKI after CABG surgery were identified. In this study, the Scr in the first 24 h after surgery and the last preoperative Scr level were recognized as the most important predictor variables. As an accepted laboratory indicator for the diagnosis of kidney injury, the preoperative Scr is reportedly the key predictor of cardiac surgery-associated AKI with ML algorithms [34, 39]. Preoperative eGFR as an independent risk factor for AKI after CABG surgery has been confirmed by some studies [3, 40, 41]. Charat et al. listed eGFR as an important risk factor in their ML model for predicting postoperative AKI of cardiac surgery [42]. Nevertheless, more research is needed to determine if body surface area (BSA) is an independent risk factor for postoperative AKI of CABG surgery, although there is no doubt that eGFR, Scr, and BSA are inextricably linked. In fact, BSA and Scr are incorporated in the Cockcroft-Gault (CG) equation for estimating eGFR [43]. Our previous study shows that the CG equation has significantly high discriminatory power to predict in-hospital mortality in patients undergoing CABG [8]. Therefore, there is logical reason to believe that these factors also play important roles in the occurrence of postoperative AKI. Notably, pulmonary arterial hypertension (PAH) is ranked as the fourth significant variable. PAH is an important risk factor for AKI after transcatheter aortic valve implantation [44] and is closely related to right heart function [45, 46]. Reportedly, right ventricular failure is associated with severe postoperative AKI of cardiac surgery [47] and PAH is one of the top five risk factors, providing a new viewpoint on clinical decision making.

GBDT (Gradient Boosting Decision Tree), a long-lasting model in ML, mainly aims to use weak classifiers (decision trees) to iteratively train and get the optimal model that has good training effect and less overfitting. LightGBM, a framework to implement GBDT, supports efficient parallel training, and has faster training speed, lessr memory consumption, higher accuracy, supporting distributed and rapid processing of massive data [15]. For the first time, this study utilized LightGBM to develop prediction models of AKI after CABG surgery. It achieved optimal predictive performance, and outperformed the SVM, Softmax and RF models.

### Limitations

There are several limitations. First, urine output criteria are not used due to missing records, while standard diagnosis of AKI is Scr or urine output criteria. Second, there are patients with impaired GFR but normal SCr levels before the operation, which are called occult renal impairment in clinical practice [48]. Thus, relying only on Scr may lead to bias in diagnosis [49]. Third, this observational study has a long duration, so there may be factors that affect our results due to improvements in surgical techniques and perioperative care. Fourth, a noteworthy limitation of our study is the exclusion of 885 patients from JSPH, constituting a significant proportion of the registered patients. Although these exclusions were applied consistently across both medical centers, they could potentially introduce selection bias and impact the generalizability of our findings. Fifth, in this retrospective study, we did not have access to physician assessments as direct comparisons for our predictive models.

## Conclusions

This study provides a LightGBM-based predictive model that can accurately predict AKI after CABG surgery. This ML-based model shows good predictive ability in both internal and external validation. It may help cardiac surgeons to intervene early in patients undergoing CABG with high risk of AKI and reduce associated complications.

## Data Availability

We are pleased to share data. The data involved in our research are available from the corresponding author. We will respond in 7 days on reasonable request.

## Declarations

Patients provided informed consent to undergo treatment, but the requirement for informed consent for participation in the current study was exempted by the ethics committees (Ethics Committees of Jiangsu Province Hospital, Ethics Committees of Shanghai Chest Hospital, Ethics Committees of Qilu Hospital and, Ethics Committees of General Hospital of Ningxia Medical University), due to its retrospective nature.

All methods were performed in accordance with the relevant guidelines and regulations.
